# Psychological Correlates of Life Satisfaction Among Medical Students in Northern India: A Cross-Sectional Study

**DOI:** 10.7759/cureus.110580

**Published:** 2026-06-10

**Authors:** Divyansh Singh, Anish Khanna, Saurabh Kashyap, Sujita K Kar, Abhishek Singh

**Affiliations:** 1 Community Medicine and Public Health, King George's Medical University, Lucknow, IND; 2 Psychiatry, King George's Medical University, Lucknow, IND; 3 Biostatistics and Epidemiology, King George's Medical University, Lucknow, IND

**Keywords:** coping strategies, life satisfaction, medical students, meditation, psychological well-being, self-confidence, stress, subjective happiness

## Abstract

Introduction: The medical curriculum is demanding and often associated with significant stress among students. This study aimed to assess life satisfaction among undergraduate medical students in Northern India and explore factors associated with life satisfaction, including sociodemographic and psychological factors.

Methods: This cross-sectional study was conducted among undergraduate medical students from selected medical colleges in Northern India. A multistage random sampling technique was used to select institutions and participants. Data were collected using a structured questionnaire administered through confidential face-to-face interviews after obtaining informed consent. Postinterview deidentification procedures were implemented. No names or direct identifiers were recorded on the questionnaire forms, and each participant was assigned a unique study code.

Results: A total of 415 students participated, of whom 64.1% were male participants. Overall, 64.6% reported being satisfied with their lives. Life satisfaction showed a significant negative correlation with perceived stress (r = -0.331, p < 0.001) and moderate positive correlations with subjective happiness (r = 0.477, p < 0.001) and self-confidence (r = 0.411, p < 0.001). Weak positive correlations were observed between life satisfaction and meditation practice (r = 0.181), spirituality (r = 0.189), gratitude (r = 0.184), and appreciation of beauty (r = 0.227), indicating small effect sizes despite statistical significance (all p < 0.001). In multiple linear regression analysis, subjective happiness (β = 0.328, p < 0.001), self-confidence (β = 0.191, p < 0.001), and perceived stress (β = −0.170, p < 0.001) emerged as significant independent predictors of life satisfaction.

Conclusions: Subjective happiness and self-confidence were independently associated with higher life satisfaction, whereas perceived stress was independently associated with lower life satisfaction. Although coping practices such as meditation and gratitude showed weaker associations, the findings highlight the potential relevance of psychological well-being and coping-related factors in understanding life satisfaction among medical students.

## Introduction

Life satisfaction refers to an individual's overall cognitive evaluation of the quality of their life and the extent to which they perceive their goals, aspirations, and expectations to be fulfilled [1]. It is widely recognized as an important indicator of subjective well-being and quality of life and has been associated with multiple aspects of psychological health and functioning [1,2].

The psychological well-being of medical students has become an important area of interest in medical education research. Medical training is often characterized by substantial academic demands, long study hours, competitive learning environments, and exposure to emotionally challenging situations, all of which may adversely affect students' mental health and well-being [3]. Previous studies have reported high levels of stress, anxiety, burnout, sleep disturbances, and psychological distress among medical students in both international and Indian settings [4-7]. Consequently, understanding factors associated with life satisfaction among medical students is important for supporting student well-being and fostering healthier educational environments.

Historically, research on medical student well-being has focused primarily on adverse psychological outcomes such as stress, depression, burnout, and emotional distress [5-7]. Although identifying the prevalence and determinants of these conditions remains important, increasing attention has been directed toward factors that may promote well-being and life satisfaction. Positive psychological characteristics and adaptive coping resources may help individuals respond more effectively to academic and personal stressors and may contribute to improved psychological well-being [8-14].

Psychological resources such as self-confidence, subjective happiness, and positive coping practices have been associated with well-being in various populations. Similarly, practices including meditation, gratitude, spirituality, and appreciation of beauty and positive experiences have been linked to adaptive coping, emotional regulation, resilience, and psychological well-being [8-14]. The selection of these variables was informed by positive psychology literature suggesting that positive psychological characteristics and coping resources may contribute to emotional regulation, resilience, and subjective well-being [15]. Accordingly, the present study examined subjective happiness, self-confidence, gratitude, spirituality, meditation, and appreciation of beauty as potential psychological correlates of life satisfaction among medical students.

Despite growing interest in medical student well-being, comparatively fewer studies from India have simultaneously examined both distress-related factors and positive psychological characteristics in relation to life satisfaction within the same analytical framework. Most Indian studies have focused primarily on psychological distress, including stress, anxiety, depression, burnout, sleep disturbances, and academic pressure among medical students [16-19]. Therefore, the present study sought to contribute additional evidence regarding psychological factors associated with life satisfaction among undergraduate medical students in Northern India. Given the cross-sectional and exploratory nature of the study, the findings should be interpreted as associations rather than evidence of causal relationships.

The primary objective of this cross-sectional study was to assess levels of life satisfaction among undergraduate medical students in Northern India. The secondary objectives were to describe sociodemographic characteristics; examine the associations of life satisfaction with perceived stress, subjective happiness, self-confidence, gratitude, spirituality, meditation, and appreciation of beauty; and identify independent predictors of life satisfaction using multivariable regression analysis.

## Materials and methods

Study design and setting

This cross-sectional study was conducted between December 2023 and April 2024 among undergraduate medical students from selected government and private medical colleges in Northern India. The study population included MBBS undergraduate students and recently graduated interns who consented to participate in the study. Data collection was carried out during routine academic sessions and nonexamination periods to minimize the potential influence of acute examination-related stress on participants’ responses.

Sampling technique

A multistage sampling approach was employed. In the first stage, four medical colleges (two government and two private) were randomly selected from a list of National Medical Commission-recognized colleges in Uttar Pradesh. In the second stage, undergraduate MBBS students and interns from the selected institutions were invited to participate.

Although institutional selection involved randomization procedures, participant recruitment ultimately depended on student availability and willingness to participate during the data collection period. Consequently, elements of convenience sampling were present at the participant level, and selection bias cannot be excluded. The unequal representation of students across academic years may also have influenced the representativeness of the sample. Therefore, the findings should be interpreted with appropriate caution when considering generalizability to the broader medical student population.

Sample size

The sample size was initially estimated using a hypothesized prevalence of life dissatisfaction of 4.8% based on previous literature [7], assuming a 95% confidence level, an absolute precision of 2%, and a design effect of 1.5 to account for potential clustering arising from the multistage sampling approach. A finite population correction factor was additionally applied using an estimated population size of approximately 545,000 medical students, resulting in a minimum required sample size of 415 participants.

To account for potential nonresponse, a larger number of students were approached during data collection. Although the initial calculation was prevalence-based, the final sample size was also considered adequate for the correlation and multivariable regression analyses performed in the present study, given the number of predictors included in the regression model and the achieved participant-to-predictor ratio.

Data were collected using a pretested semistructured questionnaire administered through face-to-face interviews by trained researchers (see the Appendix). Face-to-face administration was selected to facilitate participation, minimize incomplete responses, and allow clarification of questionnaire items when required. Participants were informed that participation was voluntary, responses would remain confidential, and no personally identifiable information would be collected. Each participant was assigned a unique study identification code to ensure anonymity.

The questionnaire comprised three sections: sociodemographic characteristics, psychological variables and coping practices, and life satisfaction assessment. The sociodemographic section collected information on age, sex, category, religion, academic year of study, current residence, family structure, personal or family history of chronic illness, and the presence of family debt or educational loans.

The second section assessed selected psychological variables and coping practices. Subjective stress, subjective happiness, self-confidence, gratitude, spirituality, meditation, and appreciation of beauty were evaluated using researcher-developed single-item measures. Subjective happiness was defined as participants’ self-perceived emotional well-being and positive affect, whereas self-confidence referred to perceived confidence in one’s abilities and effectiveness in academic and personal functioning. Participants rated these constructs using five-point Likert-type scales. For psychological variables, response options ranged from “Very low” to “Very high,” while coping practices were assessed using frequency-based response categories ranging from “Never” to “Very often.” For statistical analyses, higher scores indicated greater levels of the respective construct; for stress, higher scores represented higher perceived stress.

These single-item measures were included to maintain questionnaire feasibility and reduce respondent burden in a large survey setting. However, they were not intended to replace validated psychometric instruments. Formal psychometric validation, construct validity assessment, and test-retest reliability testing were not performed. Consequently, these measures should be interpreted as preliminary indicators of the respective psychological constructs, and findings involving these variables should be interpreted with appropriate caution.

The third section assessed life satisfaction using the Satisfaction with Life Scale (SWLS) [20], a widely used and validated instrument consisting of five items measuring global cognitive judgments of life satisfaction. Each item is rated on a seven-point Likert scale ranging from 1 (strongly disagree) to 7 (strongly agree), yielding a total score between 5 and 35, with higher scores indicating greater life satisfaction. In the present study, the SWLS demonstrated acceptable internal consistency, with a Cronbach’s alpha coefficient of 0.787.

Prior to administration, the questionnaire was reviewed by faculty members with expertise in psychiatry, psychology, and public health to assess clarity, content relevance, and contextual appropriateness. The questionnaire was also pretested to evaluate feasibility and comprehensibility. However, formal psychometric validation was not conducted for the researcher-developed single-item measures.

Ethical considerations

Ethical approval for the study was obtained from the Institutional Ethics Committee of the study institution (Letter No. 2636/Ethics/2023; Ref. Code XVI-PGTSC-IIA/P59). Participation in the study was voluntary, and written informed consent was obtained from all participants prior to data collection. Confidentiality and privacy of participants were maintained throughout the study. The datasets generated and analyzed in the current study are not publicly available because they contain sensitive participant information, but are available from the corresponding author upon reasonable request.

Data management and statistical analysis

The collected data were entered and managed using Microsoft Excel 2019 (Microsoft Corporation, Redmond, WA) and analyzed using the Statistical Package for the Social Sciences, version 24.0 (IBM Corp., Armonk, NY). Descriptive statistics were used to summarize the data. Categorical variables were presented as frequencies and percentages.

Although Likert-type responses are ordinal, the variables were treated as approximately continuous for correlation and regression analyses because of the ordered multicategory response structure, the relatively large sample size, and the exploratory nature of the study. Normality of the continuous outcome variable (SWLS score) was assessed using graphical methods and distribution characteristics before regression analysis. Pearson correlation coefficients were used to examine linear associations between life satisfaction and psychological variables.

Multiple linear regression analysis was performed to identify independent predictors of life satisfaction. Variables entered into the regression model included subjective happiness, self-confidence, perceived stress, meditation, spirituality, gratitude, and appreciation of beauty based on theoretical relevance and prior literature. Prior to interpreting the regression model, the assumptions of linearity, homoscedasticity, residual distribution, and multicollinearity were assessed. Multicollinearity was evaluated using variance inflation factor (VIF) values, and no significant multicollinearity was detected (all VIF values < 2). Residual plots were examined to assess homoscedasticity and overall model fit.

Sociodemographic variables were described descriptively and were not included in the primary regression model because the principal objective of the study was to explore psychological correlates of life satisfaction. A p value of less than 0.05 was considered statistically significant. Correlation coefficients were interpreted as weak (0.10-0.29), moderate (0.30-0.49), or strong (≥0.50). All statistical tests were two-tailed.

## Results

Approximately 500 undergraduate medical students were approached during data collection; of these, 415 consented to participate and completed the questionnaire, yielding a response rate of 83%. The mean age of the participants was 23 years, and 64.1% (n = 266) were male participants.

Sociodemographic characteristics

The sociodemographic characteristics of the participants are summarized in Table 1.

**Table 1:** Distribution of medical students by sociodemographic characteristics (n = 415)

Variable	Category	Value, n (%), n = 415
Age group	≤20 years	42 (10.1)
	≥21 years	373 (89.9)
Sex	Female	149 (35.9)
	Male	266 (64.1)
Academic year	1st year	9 (2.1)
	2nd year	189 (45.5)
	3rd year	193 (46.5)
	4th year	14 (3.4)
	Intern	10 (2.4)
Family history of chronic illness	Yes	85 (20.5)
	No	330 (79.5)
Current residence	Hostel	377 (90.8)
	Home	29 (7.0)
	Other	9 (2.2)
Family type	Nuclear	312 (75.2)
	Joint	103 (24.8)
Family debt/loan	Yes	110 (26.5)
	No	283 (68.2)
	Not sure	22 (5.3)

Most students were aged 21 years or older, and the sample consisted predominantly of male participants. The majority were enrolled in the second and third years of the MBBS program, while smaller proportions were from the first year, fourth year, and internship. Most participants resided in hostels and belonged to nuclear families. A substantial proportion reported a family history of chronic illness and family debt or loan.

Psychological characteristics

The distribution of psychological characteristics among participants is shown in Table 2.

**Table 2:** Psychological characteristics of participants

Variable	Category	Value, n (%), n = 415
Subjective stress	Very low	30 (7.2)
	Low	73 (17.6)
	Moderate	145 (34.9)
	High	111 (26.7)
	Very high	56 (13.5)
Self-confidence	Very low	15 (3.6)
	Low	37 (8.9)
	Moderate	133 (32.0)
	High	143 (34.5)
	Very high	87 (21.0)
Subjective happiness	Very low	12 (2.9)
	Low	43 (10.4)
	Moderate	126 (30.4)
	High	155 (37.3)
	Very high	79 (19.0)

Table 2 suggests that although moderate stress was the single most commonly reported category, a substantial proportion of participants (40.2%, n = 167) reported high or very high levels of subjective stress, indicating a considerable psychological burden within the study population. Self-confidence levels were predominantly high (34.5%, n = 143) or moderate (32.0%, n = 133) among participants. Similarly, the majority of students reported high (37.3%, n = 155) or moderate (30.4%, n = 126) levels of subjective happiness.

Coping practices

The coping practices reported by participants are presented in Table 3.

**Table 3:** Coping practices among medical students

Practice	Category	Value, n (%), n = 415
Meditation practice	Never	42 (10.1)
	Rarely	108 (26.0)
	Sometimes	139 (33.5)
	Often	83 (20.0)
	Very often	43 (10.4)
Gratitude practice	Never	4 (1.0)
	Rarely	18 (4.3)
	Sometimes	109 (26.3)
	Often	127 (30.6)
	Very often	157 (37.8)
Spirituality practice	Never	20 (4.8)
	Rarely	40 (9.6)
	Sometimes	147 (35.4)
	Often	135 (32.5)
	Very often	73 (17.6)
Appreciation of beauty	Never	6 (1.4)
	Rarely	25 (6.0)
	Sometimes	133 (32.0)
	Often	172 (41.4)
	Very often	79 (19.0)

When combining the ‘often’ and ‘very often’ response categories, gratitude practices were the most frequently reported coping mechanism (68.4%, n = 284), followed by appreciation of beauty (60.4%, n = 251), spirituality (50.1%, n = 208), and meditation practices (30.4%, n = 126).

Distribution of life satisfaction

The distribution of life satisfaction among participants, as measured by the SWLS, is shown in Table 4.

**Table 4:** Distribution of the medical students by Satisfaction with Life Scale SWL: Satisfaction with Life

SWL category	Value, n (%), n = 415
Extremely dissatisfied	2 (0.5)
Dissatisfied	26 (6.3)
Slightly dissatisfied	57 (13.7)
Neutral	62 (14.9)
Slightly satisfied	126 (30.4)
Satisfied	105 (25.3)
Extremely satisfied	37 (8.9)
Total	415 (100)

Table 4 suggests that overall, 64.6% (n = 268) of students reported being satisfied with their lives, including 30.4% (n = 126) slightly satisfied, 25.3% (n = 105) satisfied, and 8.9% (n = 37) extremely satisfied. In contrast, 20.5% (n = 85) of students reported dissatisfaction with life, while 14.9% (n = 62) reported neutral levels of life satisfaction.

Correlation between life satisfaction and psychological variables

The correlations between life satisfaction and psychological variables are presented in Table 5.

**Table 5:** Correlation matrix between life satisfaction and psychological variables ^*^p < 0.05 ^**^p < 0.01

Variable	1. Life satisfaction	2. Spirituality	3. Gratitude	4. Appreciation of beauty	5. Meditation	6. Self-confidence	7. Subjective happiness	8. Subjective stress
1. Life satisfaction	1	-	-	-	-	-	-	-
2. Spirituality	0.189^**^	1	-	-	-	-	-	-
3. Gratitude	0.184^**^	0.244^**^	1	-	-	-	-	-
4. Appreciation of beauty	0.227^**^	0.309^**^	0.447^**^	1	-	-	-	-
5. Meditation	0.181^**^	0.049	0.052	0.058	1	-	-	-
6. Self-confidence	0.411^**^	0.309^**^	0.257^**^	0.267^**^	0.189^**^	1	-	-
7. Subjective happiness	0.477^**^	0.205^**^	0.246^**^	0.252^**^	0.238^**^	0.431^**^	1	-
8. Subjective stress	-0.331^**^	-0.095	-0.158^**^	-0.111^*^	-0.451^**^	-0.332^**^	-0.291^**^	1

Pearson correlation analysis was performed to examine the relationships between life satisfaction and psychological variables (Table 5). Life satisfaction showed a moderate negative correlation with perceived stress (r = −0.331, p < 0.001), indicating that higher stress levels were associated with lower life satisfaction.

Moderate positive correlations were observed between life satisfaction and subjective happiness (r = 0.477, p < 0.001) and self-confidence (r = 0.411, p < 0.001). Weak but statistically significant positive correlations were found between life satisfaction and meditation practice (r = 0.181, p < 0.001), spirituality practice (r = 0.189, p < 0.001), gratitude practice (r = 0.184, p < 0.001), and appreciation of beauty (r = 0.227, p < 0.001).

The correlation matrix also showed interrelationships among psychological variables. Subjective happiness was positively correlated with self-confidence (r = 0.431, p < 0.001), while gratitude was strongly correlated with appreciation of beauty (r = 0.447, p < 0.001).

Multiple linear regression analysis

The results of the multiple linear regression analysis predicting life satisfaction are shown in Table 6.

**Table 6:** Multiple linear regression predicting life satisfaction Model statistics: R² = 0.308; Adjusted R² = 0.296; F = 25.866; p < 0.001

Predictor	β	t	p
Subjective happiness	0.328	6.891	<0.001
Self-confidence	0.191	3.886	<0.001
Subjective stress	-0.170	-3.508	<0.001
Meditation	-0.015	-0.326	0.745
Spirituality	0.028	0.631	0.528
Gratitude	-0.011	-0.233	0.816
Appreciation of beauty	0.072	1.493	0.136

The overall regression model was statistically significant (F = 25.866, p < 0.001) and explained 30.8% of the variance in life satisfaction scores (R² = 0.308; adjusted R² = 0.296). The reported R² value corresponds to the full multivariable regression model including all entered predictors.

Higher subjective happiness scores (β = 0.328, p < 0.001) and self-confidence scores (β = 0.191, p < 0.001) were independently associated with higher life satisfaction scores, whereas higher perceived stress scores (β = -0.170, p < 0.001) were associated with lower life satisfaction scores. Among the included variables, subjective happiness demonstrated the strongest independent association with life satisfaction scores.

Meditation practice, spirituality, gratitude, and appreciation of beauty were not significant independent predictors after adjustment for the other psychological variables included in the model. Multicollinearity diagnostics showed no significant multicollinearity among predictors (all VIF values < 2).

## Discussion

The present study examined life satisfaction and its psychological correlates among undergraduate medical students in Northern India. Approximately 64.6% of participants reported being satisfied with their lives, although a substantial proportion also reported moderate-to-high levels of perceived stress. These findings highlight the complex psychological environment of medical education, in which life satisfaction may coexist with significant psychological burden.

Consistent with previous literature [21], perceived stress demonstrated a significant negative association with life satisfaction [22]. More than 40% of participants reported high or very high levels of stress, reflecting the substantial academic and psychological pressures experienced during medical training. Similar findings have been reported in Indian studies documenting high levels of stress, emotional exhaustion, and burnout among medical students [16-20]. A longitudinal study among Norwegian physicians also reported that work-related stress was associated with lower life satisfaction [23]. Collectively, these findings suggest that stress may represent an important correlate of life satisfaction among medical students.

In the present study, subjective stress also showed a significant negative correlation with subjective happiness, self-confidence, gratitude, appreciation of beauty, and meditation. A notable proportion of participants also reported family debt or financial obligations. Financial strain has previously been associated with increased psychological distress, stress, and reduced well-being among medical students and may represent an additional psychosocial burden during medical training. Although financial variables were not included in the primary regression analysis, their potential influence on student well-being warrants further investigation in future research.

Subjective happiness and self-confidence showed significant positive associations with life satisfaction. These findings are consistent with previous research suggesting that positive emotional well-being, self-esteem, and self-confidence are associated with greater life satisfaction and psychological resilience [24,25]. Furthermore, multiple regression analysis identified subjective happiness as the strongest independent predictor of life satisfaction, followed by self-confidence and perceived stress. These findings suggest that internal psychological characteristics may have stronger associations with life satisfaction than individual coping practices alone.

Positive coping practices, including spirituality, gratitude, meditation, and appreciation of beauty, were also positively associated with life satisfaction. However, the observed correlation coefficients for these variables were relatively small (r = 0.181-0.227), indicating weak effect sizes despite statistical significance. These findings should therefore be interpreted cautiously and viewed as exploratory associations. Furthermore, none of these variables remained significant independent predictors in the multivariable regression model after adjustment for subjective happiness, self-confidence, and perceived stress. This suggests that although such practices may contribute to psychological well-being, their independent association with life satisfaction appears comparatively modest within the present study. The relatively small effect sizes should also be interpreted in the context of the exploratory single-item measures used to assess several psychological constructs.

An interesting observation was the higher frequency of gratitude and spirituality-related practices compared with meditation. This difference may reflect the greater integration of gratitude and spirituality into routine daily life, whereas meditation may require more structured and intentional engagement. Previous studies have similarly reported positive associations between coping strategies, psychological well-being, and life satisfaction [8-11].

The present study also demonstrated interrelationships among positive psychological characteristics. Gratitude was positively associated with appreciation of beauty, while subjective happiness was positively associated with self-confidence. These findings suggest that positive psychological characteristics may cluster together and collectively contribute to psychological well-being [26].

The findings may have implications for medical student well-being initiatives. However, given the cross-sectional design and exploratory nature of several measures, the results should be interpreted cautiously. Future studies using longitudinal designs and validated psychometric instruments are needed to further clarify the relationships identified in the present study.

Overall, the findings suggest that life satisfaction among medical students may be associated with a combination of psychological characteristics and coping-related factors. However, given the cross-sectional design and the exploratory nature of several measures, these associations should be interpreted cautiously and should not be regarded as evidence of causal relationships.

Strengths and limitations

This study has several strengths. First, it included participants from multiple medical institutions, including both government and private colleges, thereby enhancing the diversity of the study sample. Second, life satisfaction was assessed using the SWLS, a widely used and validated instrument with acceptable internal consistency in the present study. Third, the study examined life satisfaction in relation to multiple psychological characteristics and coping-related factors, allowing a broader assessment of potential correlates of life satisfaction among medical students. Finally, the sample size was adequate for the correlation and multivariable analyses performed and included students from different institutional settings within Northern India.

Several limitations should also be acknowledged. First, the cross-sectional design precludes causal or temporal interpretation of the observed associations. Second, several psychological constructs, including subjective stress, subjective happiness, self-confidence, gratitude, spirituality, meditation, and appreciation of beauty, were assessed using researcher-developed single-item measures rather than validated psychometric instruments. Because formal psychometric validation, construct validity assessment, and test-retest reliability testing were not performed, measurement precision may have been limited, and findings involving these variables should be interpreted cautiously, particularly when comparing results with studies using validated psychometric instruments.

Third, although institutions were selected using a multistage sampling approach, participant recruitment ultimately depended on student availability and willingness to participate. Therefore, elements of convenience sampling were present at the participant level, and selection bias cannot be excluded. The unequal representation of students across academic years may also have affected the sample's representativeness and limited generalizability to the broader medical student population.

Fourth, interviewer-administered self-reported questionnaires may have been subject to recall bias and social desirability bias despite assurances of confidentiality. Finally, residual confounding from unmeasured academic, social, environmental, and personal factors may have influenced the observed associations.

Despite these limitations, the study provides exploratory insights into psychological factors associated with life satisfaction among medical students and may help inform future research in this area.

Implications and future research

The findings suggest that psychological well-being and perceived stress may be relevant factors in understanding life satisfaction among medical students. However, given the modest effect sizes observed for several coping-related variables and the exploratory nature of some measures, these findings should be interpreted cautiously.

Future studies should employ validated psychometric instruments, longitudinal study designs, and more representative sampling approaches to better clarify the relationships between psychological characteristics, coping practices, and life satisfaction among medical students. Additional research across different geographic and sociocultural settings within India may further improve understanding of factors associated with student well-being [27].

## Conclusions

This cross-sectional study explored life satisfaction and its psychological correlates among undergraduate medical students in Northern India. Although a majority of participants reported moderate-to-high levels of life satisfaction, a substantial proportion also reported moderate to high levels of perceived stress. Life satisfaction was positively associated with subjective happiness, self-confidence, and several coping-related practices, whereas perceived stress demonstrated a negative association with life satisfaction.

In multivariable analysis, subjective happiness, self-confidence, and perceived stress were independently associated with life satisfaction, while the associations observed for spirituality, gratitude, meditation, and appreciation of beauty were comparatively modest and did not remain significant after adjustment. These findings suggest that psychological well-being may be associated with life satisfaction among medical students; however, the observed relationships should be interpreted cautiously given the cross-sectional design, the use of several exploratory single-item measures, and the potential for selection bias.

Future studies employing longitudinal designs, validated psychometric instruments, and more representative sampling approaches are needed to further clarify the relationships between psychological characteristics, coping practices, and life satisfaction among medical students.

## Appendices

Questionnaire used in the study

The data for the present study were collected using a structured questionnaire developed for the purpose of this research. The questionnaire included items assessing sociodemographic characteristics, psychological experiences, coping practices, and life satisfaction.

Some items were adapted from commonly used constructs in psychological research, while others were developed by the authors to capture relevant experiences among medical students. The questionnaire was reviewed for clarity and content relevance before administration.

Participants responded using Likert-type response scales appropriate for Sections B, C, and D.

Section A: Socio-Demographic Information

1. Age (in years): _________

2. Sex

o   Male

o   Female

3. Academic Year of MBBS

o   1st Year

o   2nd Year

o   3rd Year

o   4th Year

o   Intern

4. Current Residence

o   College Hostel

o   Home

o   Rented House/Apartment

o   Other

5. Type of Family

o   Nuclear Family

o   Joint Family

6. Family History of Chronic Illness (including psychiatric illness)

o   Yes

o   No

7. Family Debt or Loan

o   Yes

o   No

o   Not sure

Section B: Psychological Characteristics

Participants responded to the following statements using a five-point scale (1-5) reflecting the frequency of their experiences or their level of agreement.

1. How often do you feel that important aspects of your life, such as academic responsibilities or personal relationships, are difficult to manage or control?

Response options ranged from:

o   1 = Very rarely

o   2 = Rarely

o   3 = Sometimes

o   4 = Often

o   5 = Very often

2. How often do you feel that the challenges or pressures in your life become overwhelming or difficult to handle?

Response options ranged from:

o   1 = Very rarely

o   2 = Rarely

o   3 = Sometimes

o   4 = Often

o   5 = Very often

3. At present, I feel confident about myself and believe that I possess several positive qualities and abilities.

Response options ranged from:

o   1 = Strongly disagree

o   2 = Disagree

o   3 = Neutral

o   4 = Agree

o   5 = Strongly agree

4. In general, I consider myself to be a happy or cheerful person in everyday life.

Response options ranged from:

o   1 = Strongly disagree

o   2 = Disagree

o   3 = Neutral

o   4 = Agree

o   5 = Strongly agree

Section C: Coping Practices

Participants indicated how frequently they engaged in the following behaviors.

1. How often do you practice meditation, mindfulness, or other activities intended to calm the mind and improve focus?

2. How often do you engage in spiritual or religious practices in your daily life?

3. When someone supports or helps you, how often do you consciously express gratitude or appreciation?

4. How often do you experience strong feelings of wonder, appreciation, or emotional connection when encountering beautiful scenes, music, nature, or art?

Response options for all items:

o   Never

o   Rarely

o   Sometimes

o   Often

o   Very Often

Section D: Satisfaction With Life Scale (SWLS)

Life satisfaction was assessed using the Satisfaction With Life Scale (SWLS) consisting of five statements.

1. In most ways‚ my life is close to my ideal.

2. The conditions of my life are excellent.

3. I am satisfied with my life. 

4. So far‚ I have gotten the important things I want in life.

5. If I could live my life over‚ I would change almost nothing.

Participants indicated their agreement using a seven-point Likert scale:

o   1 = Strongly disagree

o   2 = Disagree

o   3 = Slightly disagree

o   4 = Neither agree nor disagree

o   5 = Slightly agree

o   6 = Agree

o   7 = Strongly agree
